# Outbreak of *Clostridium perfringens* food poisoning linked to leeks in cheese sauce: an unusual source

**DOI:** 10.1017/S095026882000031X

**Published:** 2020-02-27

**Authors:** Alex Bhattacharya, Saran Shantikumar, Damon Beaufoy, Adrian Allman, Deborah Fenelon, Karen Reynolds, Andrea Normington, Musarrat Afza, Dan Todkill

**Affiliations:** 1 Field Epidemiology Training Fellow, North West Field Service, Public Health England, London, UK; 2 Specialist Registrar, FS West Midlands, Public Health England, London, UK; 3 Environmental Health Practitioner, Shropshire Council, Shropshire, UK; 4 Chartered Environmental Health Practitioner, Shropshire Council, Shropshire, UK; 5 Out-posted Scientist, Food Water & Environmental Microbiology Services, West Midlands, Public Health England, London, UK; 6 Public Health Services Manager, Public Health Laboratory Birmingham, Heart of England NHS Foundation Trust, London, UK; 7 Health Protection Nurse, West Midlands Health Protection Team, Public Health England, London, UK; 8 Consultant in Communicable Disease Control, West Midlands Health Protection Team, Public Health England, London, UK; 9 Consultant Epidemiologist, FS West Midlands, Public Health England, London, UK

**Keywords:** Cheese, *Clostridium perfringens*, leeks, outbreak

## Abstract

Between 11–13 December 2018, local public health authorities in the West Midlands, England were alerted to 34 reports of diarrhoea with abdominal cramps. Symptom onset was ~10 h after diners ate Christmas meals at a restaurant between 7–9 December 2018. A retrospective case-control study, environmental and microbiological investigations were undertaken to determine the source and control the outbreak. An analytical study was undertaken with odds ratios (OR) and 95% confidence intervals (CI). Forty persons were recruited to the analytical study (28/40 cases). Multivariable analysis found that leeks in cheese sauce was the only item associated with illness (aOR 51.1; 95% CI 4.13–2492.1). Environmental investigations identified significant lapses in food safety, including lapses in temperature control during cooking and hot holding, likely cross-contamination between raw and cooked foods and the reuse of leftover cheese sauce for the next day's service. No food samples were taken during the exposure period. Two faecal samples were positive for *Clostridium perfringens* with one confirming the enterotoxigenic gene. Cheese sauce is an unusual vehicle for the organism and the first time this has been reported in England.

## Introduction

*Clostridium perfringens* is a Gram-positive, spore-forming bacterium which causes illness in humans through the production of toxins. Naturally occurring in the environment, soil, water and in the gut flora of humans and animals, *C. perfringens* is a common source of food poisoning in the United Kingdom [1, 2]. Ingestion of toxin-producing *C. perfringens* results in food poisoning, with an incubation period between 5–24 h, commonly associated with diarrhoea and abdominal pain [2]. Symptoms are often self-limiting within 24 h, as a result it is expected that most cases are not reported [1, 3]. The United Kingdom (UK) infectious intestinal disease 2 study estimated 90 000 *C. perfringens* cases per year, with an incidence of 1.5/1000 person years; however, only 17% of *C. perfringens* cases were estimated to present to their general practitioner [4]. In England, it is estimated that 8–13% of gastrointestinal foodborne outbreaks are associated with *C. perfringens* [2, 5, 6].

Growth of *C. perfringens* occurs at temperatures of 12–54 °C which may occur during cooling, reheating and hot holding of cooked foods [1]. Enterotoxigenic *C. perfringens* spores are highly resistant to cooking and remain dormant at low temperatures; spores germinate at temperatures up to 50 °C with a growth range between 15–55 °C (optimum 43–47 °C) [7–9]. The UK Food Standards Agency (FSA) recommends cooking food until it has reached an internal temperature of 70 °C [10].

Between 11–13 December 2018, local public health authorities were alerted to 34 reports of diarrhoea with abdominal cramps from diners who ate Christmas meals at a restaurant between 7–9 December 2018 in the West Midlands, England. An outbreak was declared and an outbreak control team (OCT) was convened on 14 December 2018 to undertake an investigation to determine the source and control the outbreak. This paper describes the investigation, findings and public health action resulting from the outbreak.

## Methods

### Epidemiological investigations

Eight individuals reported illness to the FSA or to Shropshire Council on behalf of their dining group after members of their group became unwell following dining at the restaurant between the 7th–9th December. The reporting individuals will hereafter be referred to as the group organiser. Group organisers provided summary figures for how many were in their group, became unwell, and some information on symptoms and onset. Cases were defined as persons who had eaten at the restaurant between the 7th–9th December, followed by any symptoms of gastroenteritis with a date of onset following dining. Controls were defined as persons who had eaten at the restaurant between the 7th–9th December and had not developed any symptoms of gastroenteritis.

An online questionnaire was developed using Public Health England's (PHE) SelectSurvey system to capture self-reported outcome and exposure information. The questionnaire asked persons about symptoms of gastroenteritis, date of onset, duration of symptoms, severity, travel and household sickness prior to the event. Exposure information on foods consumed whilst dining at the restaurant was captured. The group organiser for each group was re-contacted, interviewed with option of completing the questionnaire over the phone, sent an email or SMS with a link to the online questionnaire and asked to cascade the message electronically to the others from their dining groups. Recruitment took place between 19th–24th December 2018. SMS messages were sent using the web service MessageMedia [11].

Descriptive epidemiology was undertaken using the aggregate data provided by the group organiser. A retrospective case-control study was undertaken using the self-reported dining groups as a sampling frame. Demographics and exposures among study participants were described and compared using the questionnaire data. Univariable and multivariable analysis was undertaken. Stratified analysis was undertaken to investigate confounding using differences between crude and adjusted Mantel–Haenszel (MH) odds. We fitted logistic regression models to estimate adjusted measures of association between exposures and illness (odds ratios (ORs) with 95% confidence intervals (CIs)). Each menu exposure item with a *P*-value <0.1 from the univariable analysis was included in the multivariable model alongside *a priori* confounders of age group and sex. Menu item and geographical confounders were included if they significantly improved model fit, as measured using Akaike's Information Criterion, in the final models.

Data analysis was conducted in R v3.5.1 [12]; the R package ‘EpiStats’ v1.2 [13] was used for univariable and stratified analysis; the base package ‘stats::glm’ [12] was used for multivariable analysis.

### Environmental investigations

Shropshire Council Environmental Health Practitioners (EHPs) conducted four site visits during the investigation. During site visits EHPs gathered information on food hygiene, cleanliness and food safety management systems (Hazard Analysis and Critical Control Point Analysis (HACCP)) and staff training. Eight food samples were taken and submitted for analysis on the 14th December from what was available at the time of the visit; no samples of the food were taken during the putative exposure period.

### Microbiological investigations

Faecal samples were collected from five cases. All samples were tested for *Clostridium perfringens*, *Shigella* sp., *Escherichia coli* O157, *Salmonella* sp., *Campylobacter* sp. and *Bacillus cereus* by culture, and Norovirus by the polymerase chain reaction (PCR) at the Birmingham Public Health laboratory with confirmation testing at the PHE gastrointestinal bacteria reference unit.

All food samples were analysed at PHE Food Water Environmental Laboratories and were tested for aerobic colony counts, *Clostridium perfringens*, *Shigella* sp., *Escherichia coli* O157, *Salmonella* sp., *Listeria* sp., *Campylobacter* sp., *Bacillus cereus* by culture, and, Norovirus by the PCR.

## Results

### Epidemiological investigations

#### Self-reported dining groups

In total, there were 102 diners across the groups (group size range 1–41; Table 1). Of the 102 reported diners who had eaten at the restaurant, 44 were reported to have no symptoms whereas 58 were reported unwell. Onset dates were only available as estimates for 20 cases as numbers ill and onset were provided by the group organiser and not directly from the cases in each dining group; median onset was 9.75 h (Table 1). Across the weekend, one group of five diners (Group E) ate at the dinner service which was prepared during the day; the remaining 97 diners ate at the lunch service which had been prepared overnight. Shropshire Council was made aware of four additional cases among the staff at the restaurant; however, they were excluded from the analytical study as we could not establish food exposures or exposure dates.

**Table 1.** Dates and times of exposure and onset for self-reported dining groups

Group	Dining group size (*n*)	Reported symptomatic (*n*)	Dining reservation		Onset of symptoms	
			Date	Time	Reported onset	Estimate onset^a^ (median)
A	41	14	08 Dec 2019	12:00	1	15.5 h
B	17	12	09 Dec 2019	12:30	1	11 h
C	17	11	09 Dec 2019	12:00	1	12 h
D	16	10	08 Dec 2019	13:00	10	9.5 h
E	5	5	09 Dec 2019	18:00	0	
F	3	3	07 Dec 2019	12:30	1	10 h
G	2	2	07 Dec 2019	12:45	2	6.5 h
H	1	1	09 Dec 2019	12:30	1	15 h
Total	102	58			20	9.75 h

a Where onset estimates were reported as a range, the midpoint was used.

#### Analytical study

**Fig. 1.**
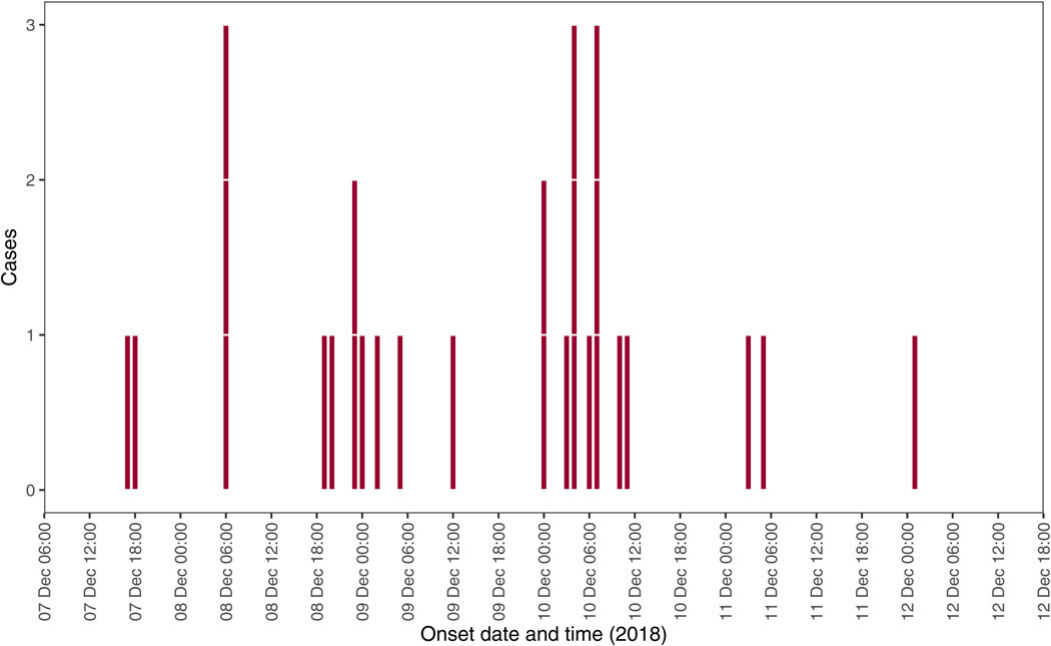
Epidemic curve by date and time of onset reported among cases, West Midlands, England, Dec 2018 (*n* = 28).

Of the possible 102 individuals identified by the group organisers, 43 people responded to the questionnaire (39.2% response rate). Three responders did not meet the case definitions, as they did not eat at the restaurant, and were removed from the analysis. Of the remaining respondents, 28 were cases (70%), 12 were controls (30%). The median age of respondents was 65 years (interquartile range (IQR) 48–73 years) with a range of 5 to 86 years old. Sixty-three percent of cases were female. Except for two cases, all ate during the lunch services (meal starting between 12:00–14:00). The epidemic curve indicates a point source (3-days of exposure) outbreak with a peak in onset of cases illness on Monday 10 December (Fig. 1).

Of the 28 cases, 27 experienced diarrhoea and abdominal pain (96.4%), and 4 reported vomiting (14%). One case reported hospitalisation. The median incubation period was 17 h (IQR 11–35 h) with a range of <1 h to 3.4 days. Half of the cases (53%) reported symptoms lasting less than 24 h, and 79% reported symptoms lasting 48 h or less.

The carvery side buffet included: stuffing, gravy, Yorkshire pudding, roast potatoes, new potatoes, mashed swede, carrots, parsnips, broccoli, brussels sprouts, peas, leeks in cheese sauce, cauliflower cheese, cranberry sauce, mustard, horseradish and/or mint sauce. Diners could also choose a carvery roast meat (beef, pork and/or turkey), or a main from a la carte menu. There was no significant difference in onset of illness and attendance date across the weekend (*P* = 0.18). Odds of illness by exposures can be found in Table 2.

**Table 2.** Univariate analysis of food exposures among cases and controls (*n* = 40)

Exposure	Cases			Controls			OR	95% CI		*P*-Value
	*N*	*n*	%	*N*	*n*	%		Lower	Upper	
Leeks in cheese sauce	28	23	82.14	12	3	25.00	13.80	2.19	100.50	0.001
Mashed swede	28	12	42.86	12	1	8.33	8.25	0.91	383.33	0.063
Stuffing	28	14	50.00	12	2	16.67	5.00	0.81	53.05	0.079
Cauliflower cheese	28	17	60.71	12	3	25.00	4.64	0.86	31.34	0.082
Horseradish sauce	28	4	14.29	12	0	0.00	Inf	0.28	Inf	0.297
Roast potatoes	28	23	82.14	12	12	100.00	0.00	0.00	2.49	0.298
Beef	28	8	28.57	12	5	41.67	0.56	0.11	2.99	0.476
Parsnips	28	18	64.29	12	6	50.00	1.80	0.37	8.76	0.49
Pork	28	13	46.43	12	4	33.33	1.73	0.35	9.65	0.505
Gravy	28	22	78.57	12	11	91.67	0.33	0.01	3.39	0.652
Carrots	28	20	71.43	12	10	83.33	0.50	0.04	3.28	0.693
Yorkshire pudding	28	19	67.86	12	9	75.00	0.70	0.10	3.86	0.725
Sprouts	28	15	53.57	12	5	41.67	1.62	0.34	8.09	0.731
Turkey	28	16	57.14	12	6	50.00	1.33	0.28	6.40	0.738
Cranberry sauce	28	9	32.14	12	4	33.33	0.95	0.19	5.48	1
Mustard sauce	28	2	7.14	12	1	8.33	0.85	0.04	54.41	1
Mint sauce	28	2	7.14	12	1	8.33	0.85	0.04	54.41	1
Peas	28	17	60.71	12	8	66.67	0.77	0.14	3.83	1
New potatoes	28	7	25.00	12	3	25.00	1.00	0.17	7.35	1
Broccoli	28	8	28.57	12	3	25.00	1.20	0.21	8.63	1

OR, odds ratio; CI, confidence interval.

Four reported food exposures were of interest from the univariable analysis, with increased odds of illness and *P* < 0.1, compared to not being exposed to that menu item: leeks in cheese sauce (OR 13.8; 95% CI 2.2–100.5), mashed swede (OR 8.3; 95% CI 0.9–383.3), stuffing (OR 5.0; 95% CI 0.8–53.1) and cauliflower cheese (OR 4.6; 95% CI 0.9–31.4) (Table 2). Of these, only leeks in cheese sauce were statistically significant, but all four exposures were chosen for inclusion in further analysis. With a shared ingredient, cauliflower cheese is a probable confounder, and was found to be an effect modifier for leeks in cheese sauce (MH aOR for leeks in cheese sauce adjusted for cauliflower cheese = 12.0 95% CI 1.8–81.8; 13% change). Cauliflower cheese was included in the final multivariable model alongside *a priori* confounders, age and sex. Almost half the diners (45%) who ate cheese dishes ate both cauliflower cheese and leeks in cheese sauce from the carvery buffet, and 70% of them ate at least one cheese dish.

After adjustment for other exposures of interest and confounding, the odds of becoming ill were 50 times higher (aOR = 51.1; 95% CI 4.13–2492.1; *P* = 0.01) in those who consumed leeks in cheese sauce compared to those who did not consume the dish (Table 3).

**Table 3.** Multivariable analysis of exposures among cases and controls (*n* = 40)

Exposure	aOR	95% CI low	95% CI high	*P*-Value
Leeks in cheese sauce	51.12	4.13	2492.10	0.01
Stuffing	22.28	1.52	1482.33	0.06
Mashed swede	12.06	0.87	517.61	0.11
Age	1.03	0.97	1.12	0.33
Cauliflower cheese	0.46	0.03	4.87	0.53
Sex (male)	0.40	0.04	3.54	0.41

aOR, adjusted odds ratio; CI, confidence interval.

### Environmental investigations

The first visit on the 11th December 2018 highlighted lapses in standard food hygiene and temperature control during cooking, hot holding and serving. A member of bar staff was reported working whilst symptomatic and a member of the public was allegedly sick on the premises the day before the first reported exposure. The food hygiene history of the premises was previously judged as ‘very good’, consistently maintaining a food hygiene rating of ‘5’ out of 5 over the past 7-years. Reports from previous ratings highlighted minor concerns regarding non-compliance for structural and cleaning issues. Three additional visits were undertaken by the EHPs, on the 14th, 15th and 17th December 2018.

Investigations on food safety practices following the epidemiological study found that the cheese sauce used with both the leeks and cauliflower was the same, however preparation and heating of the dishes differed. The cheese sauce for the leeks and cauliflower was made from a mix of pre-packaged grated cheese and dehydrated béchamel packet sauce melted together in ~15L of hot water in a large pot. For the preparation of the cheese sauce, a large volume was made in the morning and left to cool unrefrigerated for use throughout the day. The Food Business Operator (FBO) reported that leftover cheese sauce from the evening service may have been used the next day at the lunch service. Leeks were prepared in large batches in advance, steamed, cooled down at room temperature, placed into walk-in chiller and retrieved when required. The pre-prepared cheese sauce was then added to the portion of leeks and re-heated in the microwave prior to service. The cauliflower was prepared in much smaller quantities, steamed and then immediately placed into oven pans, cheese sauce was added and then grated cheese was placed on top. This dish was then placed direct to service.

**Fig. 2.**
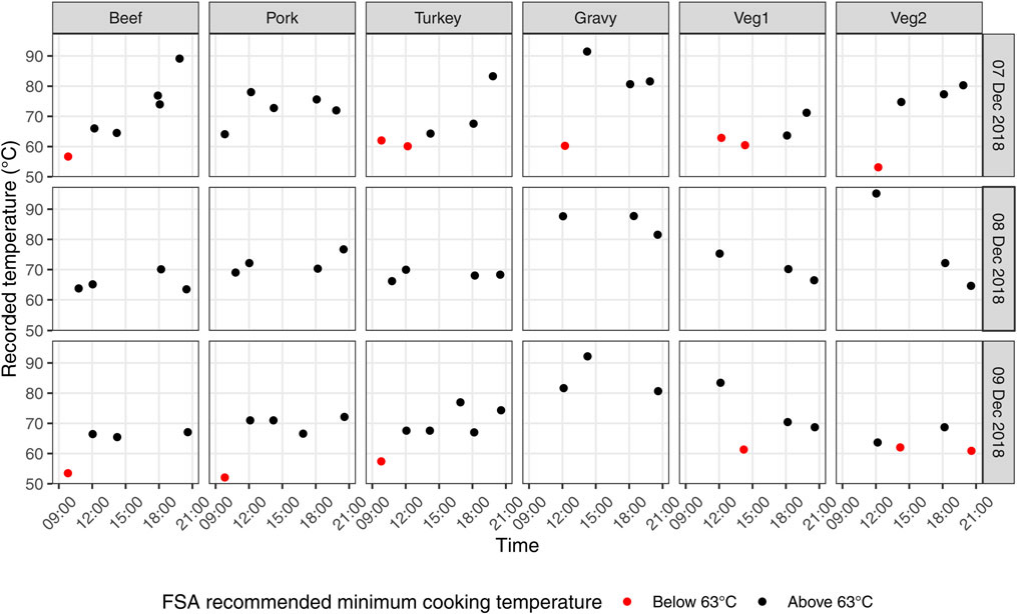
Automated recordings of food temperatures between 7–9 December 2018 of three meats, gravy and unspecified vegetables.

Before and during the outbreak period, food temperatures were being recorded via a digital temperature recording system (https://www.checkit.net/). The system was set up with predetermined food items and basic temperature range parameters. The automated temperature recordings were taken for the beef, pork, turkey, gravy and two unspecified vegetable sides (recorded as Veg1 and Veg2), as such it is unclear if the same side dishes were sampled (Fig. 2). Importantly, only the FBO had access to these recorded values, not the rest of the staff. During the investigation, a lookback into these temperatures found that internal recorded hot-holding temperatures for various dishes, notably the vegetable sides, were below the FSA recommended 63 °C. The temperature records did not clearly differentiate which were taken during cooking or hot holding. These temperatures were suboptimal to prevent the growth or proliferation of *C. perfringens.* We observe better temperature control for the evening service as food was cooked during the day while the kitchen was staffed following the lunch service and placed in hot holding for consumption immediately after cooking.

There was no evidence for the contamination of meats, however, poor temperature control was observed for the cooking of carvery meats. Large joints of beef, pork and turkey were cooked overnight in Alto-Shaam ovens with reported temperatures insufficient to prevent the growth of bacteria; meats were held in the sealed ovens at low temperatures overnight. Carvery meats were reheated for the lunchtime service in the ovens; pan juices from the oven trays were added to the gravy. We can see in Figure 2 that recorded internal temperatures of the meats prior to the lunch service (before 11 am) were frequently below 63 °C, however the meat temperatures were recorded at safe levels during serving. Likewise, pan juices would have been taken in the morning to prepare the gravy for the lunch service; except for the 7th December, gravy temperatures were routinely above 80 °C.

#### Control measures

Food practice changes were enforced by Shropshire Council. On 20th December 2018, a Hygiene Improvement Notice was served on the food business for failing to review the food safety management procedures based on the HACCP principles. Further monitoring checks were carried out during the Christmas holiday period to ensure compliance with recommended control measures. To prevent cross-contamination in the kitchen, EHPs recommended that specific preparation areas of the kitchen were designated for vegetables and other non-meat items, and that raw and cooked foods were also prepared in separate areas. Other recommendations included: safe disposal of leftovers, including cheese sauce, at the end of each day; improved electronic monitoring of all meat and vegetable dishes during cooking and in the carvery hot holding area using the existing automated system every 2-h with corrective action; replacement of oven temperature probes; use of a blast chiller for cooling processes; new hand wash basins provided for staff in the hot-hold serving area; designated separate kitchen areas for raw and cooked vegetable preparation; all staff being retrained in Level 2 Food Hygiene and HACCP training for head chefs. Leeks in cheese sauce were voluntarily removed from the menu by the FBO.

### Microbiological investigations

Food samples were taken including: two samples each of cooked turkey and cooked beef, one sample each of cooked pork, cauliflower in cheese sauce, leeks in cheese sauce and gravy. All food samples were negative for *Clostridium perfringens*, *Shigella* sp., *Escherichia coli* O157, *Salmonella* sp., *Listeria* sp., *Campylobacter* sp., *Bacillus cereus* and Norovirus. Indicator organism counts on food samples were satisfactory or borderline for all samples with the exception of the cauliflower cheese which showed an ‘unsatisfactory’ aerobic colony count (≥105 cfu/g) according to current ready-to-eat guidelines [14].

Patient stool samples were submitted by five cases on the 15th, 18th, 19th, 20th & 21st December 2018, between 9 and 12 days after the putative exposure (median 10 days). All isolates were negative for *Shigella* sp., *Escherichia coli* O157, *Salmonella* sp., *Campylobacter* sp., *Bacillus cereus* and Norovirus. *C. perfringens* was isolated from two patients. Both positive isolates were sent to the PHE reference laboratory. Both samples were confirmed as *C. perfringens* by the detection of a fragment of the alpha toxin gene, however, only one of the samples contained a fragment of the enterotoxin gene and therefore was confirmed pathogenic.

## Discussion

Based on the results of the epidemiological and environmental investigations, we concluded that this outbreak of enterotoxigenic *C. perfringens* was associated with the consumption of leftover and reheated cheese sauce. The epidemiological analysis indicated a strong association between consumption of leeks in cheese sauce and becoming ill. Food samples were taken several days after the putative exposure, and as such we lack the microbiological evidence; however, stool sample analysis confirmed enterotoxigenic *C. perfringens* as the causative organism. Environmental investigations reported food preparation methods for leeks in cheese sauce included: the reuse of leftover cheese sauce combined with ambient cooling; microwave reheating and inadequate hot-holding temperatures. As the preparation for cauliflower cheese differed, this kept the other dish with cheese sauce under temperature control. Following the removal of leeks in cheese sauce from the menu and changes to food preparation and temperature control in the kitchen and serving areas, no further cases were reported.

A review of foodborne outbreaks in England found that 80% of the reported outbreaks of *C. perfringens* cited red meat or poultry as the source, whereas only 4% reported vegetables and none reported dairy links [5]. In this atypical outbreak, environmental investigations highlighted lapses in food preparation and temperature control during the cooking of large joints of meat and whole turkeys, and observed the addition of pan juices to the gravy. However, there was no substantive evidence that meat or gravy was the cause of the outbreak. Leftover carvery meats or gravy, the usual suspects in *C. perfringens* outbreaks, were not retained for reuse. *C. perfringens* spores can be found in soil and may have been present on the leeks; these spores are notoriously hard to destroy [7, 15]. Batch steaming may have been insufficient to destroy any spores found on the leeks, allowing for germination when put in the hot holding for serving. Alternatively, raw and cooked vegetables were prepared on the same surfaces without clear sanitisation protocols; cross-contamination from raw vegetables was possible. While raw milk is a known source of *C. perfringens* outbreaks, outbreaks linked to commercially produced hard cheese are rare since pasteurisation became commonplace [16–18]. An atypical vehicle, the cheese sauce could serve as an anaerobic proteinaceous broth for the growth of *C. perfringens* over several days which may have been introduced through cross-contamination from a variety of sources, including the leeks, which are difficult to clean effectively of soil. This is the first reported instance of a *C. perfringens* outbreak associated with leeks in cheese sauce.

A limitation of the study was that we were unable to determine odds of illness by the two components of the suspect vehicle, leeks or cheese sauce. Secondly, we had several cases reporting incubation periods greater than the 24-h normally associated with *C. perfringens* (*n* = 8; median 39-h incubation); however, the symptom profile of these cases is consistent with enterotoxigenic *C. perfringens.* It would have been helpful to have timely stool samples from these individuals to aid in the investigation and support testing our hypothesis. There was difficulty in convincing cases to submit samples during the holiday period, as such we are limited by our small number of stool samples and by the delays in procuring them. Had we been able to re-contact cases, we would have explored these incubation periods and requested additional stool samples. Finally, we were unable to rule out cross-contamination from the leeks, meats or elsewhere in the kitchen as a potential source of *C. perfringens* contamination. The lack of sampling availability of any suspected food resulted in a lack of food microbiological information.

The initial epidemiological steer of the investigation meant that food samples and a full environmental investigation were not undertaken by officers. In similar circumstances, where there is limited evidence around causality, we recommend officers remain open-minded until complete verifiable epidemiological or microbiological evidence is determined. Officers were unable to view full critical control point (CCP) records, the temperature control information at the initial visit. At any similar scenarios, authorised officers, particularly in large outbreaks where initial cases are rapidly increasing, officers should focus on and carry out a full environmental audit at suspect premises and obtain and view copies of CCP records, staff training and HACCP plans as well as, in consultation with PHE, gather food samples for microbiological analysis at the earliest opportunity to identify deficiencies. Officers should consider using the full range of enforcement tools available, including Emergency Food Hygiene Prohibition Powers under Regulation 8 of The Food Safety and Hygiene (England) Regulations 2013 [19].

This outbreak also serves as a reminder to FBOs to ensure that staff training is updated when food items are added or changed on the menu especially when cooking processes change. When using digital temperature recording systems, increasingly more commonplace in food business, FBOs should ensure that multiple trained users have access and that all user-definable parameters are correctly set up for temperature CCPs and corrective limits. For example, fridges are correctly labelled and set to 8 °C or below, cook temperatures are set in accordance with the FSA Guidance and for hot holding, above 63 °C. For large batch cooking processes FBOs are also encouraged to identify and implement the correct controls using recognised rapid cooling methods, such as blast chilling, to minimise the time that food is held in the danger zone, between 8 °C and 63 °C. FBOs should also consider any additional food safety risks when vegetable dishes are combined with other nutrient rich foods, such as cheese and similar dairy-based ingredients and whether specific CCPs need to be identified, controlled and monitored in the HACCP plan.

The SMS cascade of the electronic questionnaire allowed us to undertake rapid case-finding and data collection in the week preceding Christmas enabling us to reach a wide network of people through a limited number of contacts. This method allowed a rapid collection of evidence to support the implementation of control measures. In discussion with group organisers, they reported the SMS cascade method of the online questionnaire was preferable to providing the contact information of their group members to PHE for individual follow-up. However, because of this, we could not follow-up individuals to improve response rates or clarify reported clinical information. Because the study took place during the Christmas period, it was difficult to follow up with group organisers to send reminders, however, we would recommend re-contacting the group organiser to send reminders to their groups to promote additional responses in other situations. This method would be very helpful in settings where it is difficult to gather the contact information for large numbers of people, where exposure status is unknown, for example at food markets or temporary ‘pop-up’ restaurants or events. We would recommend the use of SMS cascading for rapid dissemination of questionnaires in outbreak settings as an effective low cost method. One possible limitation of using this method is that you may be likely to get clusters of people with similarities in their exposures. In interview, some cases reported that they made similar menu choices to their respective dining groups. Using SMS to send out questionnaires has been historically successful, however, the PHE questionnaire system is not optimised for smartphones therefore some respondents may have issues with using the system on their handheld device [20].

We started this outbreak investigation with a primary hypothesis of norovirus, due to a reported staff member with suspected gastroenteritis, rapid onset of illness and poor food practices observed at the first site visit. This first hypothesis resulted in a missed opportunity, as the decision was made not to undertake food sampling. Our second hypothesis was following information on symptoms and onset, where we suspected *C. perfringens,* and focused our environmental investigation on meat and gravy preparation; these suspicions were further supported by the temperature control observations from the first site visit. Our hypotheses led us to focus the investigations, while instead we should have remained open to unusual exposures. It remains tempting to focus an investigation early particularly when time or human resources are limited, as was the case in this investigation. We would recommend keeping an open mind particularly during initial investigations and always undertaking food and environmental sampling at the first opportunity in suspected food-related outbreaks.

## Conclusions

This study found that this *C. perfringens* outbreak was associated with the consumption of leeks in cheese sauce; however, the likely vehicle was a contaminated cheese sauce. The seasonality and setting of this outbreak are in keeping with the trends of *C. perfringens* outbreaks in the UK and USA [2, 3, 8]. Improvisations in the kitchen to deal with the increased customer throughput over the Christmas, such as the use of leftovers, overnight cooking and other factors such as a lack of food preparation protocols for staff to follow may have converged to contribute to this outbreak. The rapid action and investigations were critical in rectifying breaches in food safety, providing evidence to the FBO to remove leeks in cheese sauce from the menu and taking steps to prevent further cases or other outbreaks within the restaurant over the Christmas period where public health action and respondent participation would have been more difficult.
